# The Pioneers of Homœopathy

**Published:** 1897-12

**Authors:** 


### BOOK NOTICE.

The Pioneers of Homœopathy. Compiled by Thomas Lindsley Bradford, M. D. Philadelphia: Bœricke & Tafel, 1011 Arch St. 1897. Price, cloth, $3.00 net; by mail, $3.25.

This book is designed to perpetuate the memory of the little band of workers who were identified with Hahnemann, stood loyally by him, and assisted him in his great work of creating a materia medica. This band consists first of the pioneer provers of the materia medica, "the devoted band of students who were Hahnemann's pupils in Leipzig from 1811 to 1821, and who recognized the genius of the savant, the teacher, the chemist, and the physician, and were convinced of the truth of his method of healing;" and, secondly, of the small army of practitioners who practiced Homœopathy in every country of the world, before the year 1835.

Says the author in his preface: "The members of the homœopathic school cannot know too much about the struggles, under persistent opposition, of the men who carried the law of Homœopathy into different lands; who by their devotion, their belief in its truth made it possible that the physician of our faith is to-day recognized by very many people as the exponent of the most successful and best system of medical practice."

The book is in two parts: Part I is devoted to an account of the provers who assisted Hahnemann. Part II is devoted to an account of all physicians who practiced Homœopathy before 1835.

The names are arranged in alphabetical order in both parts, and there is, in addition, an alphabetical index.

Dr. Bradford is well known for his admirable works, *Homœopathic Bibliography of the United States* and his *Life and Letters of Hahnemann*, both of which have been reviewed in these pages.

